# Supplementary material for: Direct observation of large electron–phonon interaction effect on phonon heat transport
Source: Nat Commun. 2020 Nov 27;11:6040. doi: 10.1038/s41467-020-19938-9 (PMC7695728; doi:10.1038/s41467-020-19938-9)
Supplement: Supplementary file 1 — Supplementary Information [file 41467_2020_19938_MOESM1_ESM.pdf]

Supplementary Information

Direct observation of large electron-phonon interaction effect on phonon heat transport

Jiawei Zhou[1], Hyun D. Shin[2], Ke Chen[1#], Bai Song[1§], Ryan A. Duncan[2], Qian Xu[1], Alexei A. Maznev[2], Keith A. Nelson[2], Gang Chen[1]

[1]*Department of Mechanical Engineering, Massachusetts Institute of Technology, Cambridge, MA 02139, USA;*

[2]*Department of Chemistry, Massachusetts Institute of Technology, Cambridge, MA 02139, USA*

[#]*Current address: School of Physics, Sun Yat-sen University, Guangzhou 510275, China*

[§]*Current address: Department of Energy and Resources Engineering, and Beijing Innovation Center for Engineering Science and Advanced Technology, Peking University, Beijing 100871, China*

Supplementary Figure Legends

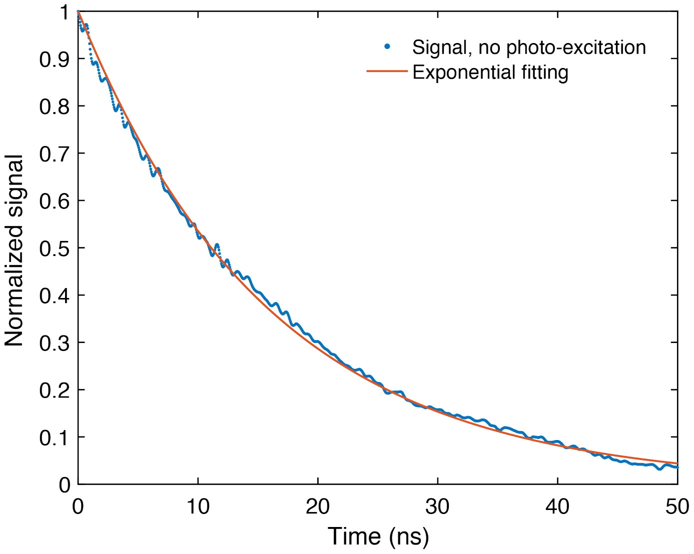

Supplementary Figure 1. Transient thermal grating signal without photo-excitation. The time zero is taken as the excitation time, and the signal is normalized to its value at time zero. The signal can be well fitted with an exponential curve given by $I_0(t) = \exp\left[-2\frac{q^2 k_{\mathrm{eff},0} t}{\rho c_{\mathrm{p}}}\right]$, where $q = \frac{2\pi}{L}$ with L being the grating period ($9\ \mu\mathrm{m}$). The fitting gives the in-plane thermal conductivity of the silicon membrane to be $\sim 105\ \mathrm{W\ m^{-1}\ K^{-1}}$.

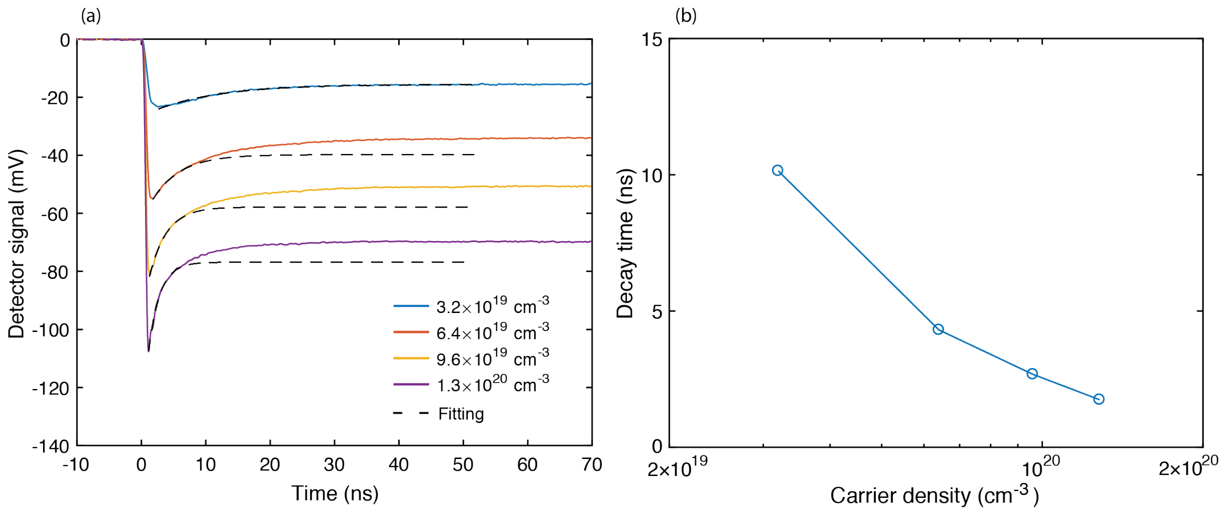

Supplementary Figure 2. Carrier recombination studies by free carrier absorption of the probe beam under photo-excitation. (a) Transmitted probe beam intensity with time under different excitation fluences. The excitation time is taken as the time zero. The excitation fluences have been converted to the corresponding initial carrier density as described in Methods. The initial decay is due to carrier recombination, while the plateau seen at later times is due to sample heating. The transient transmission signals are fitted with a single exponential decay with a constant term ($ae^{-bt} + c$). For the lowest carrier density, the fitting range is from 0 ns to 50 ns, while for all other carrier densities the fitting range is from 0 ns to 5 ns. Fitting results are plotted up to 50 ns. A single exponential decay cannot fit the entire signal well because the recombination rate changes with the carrier density. Nonetheless, the initial decay of the carrier density can be approximately described by a single exponential curve (dashed line), from which a decay time can be extracted. (b) The decay time constant corresponding to the initial carrier recombination extracted from (a). This decay time constant can be regarded as an average recombination lifetime for electrons and holes during the initial course of measurement when carriers have most significant impact on the thermal transport. The extracted decay time varies from ~10 ns to ~2 ns as the carrier density increases. In the transient absorption measurement, the power of the probe beam (532 nm) is ~17 mW before the sample.

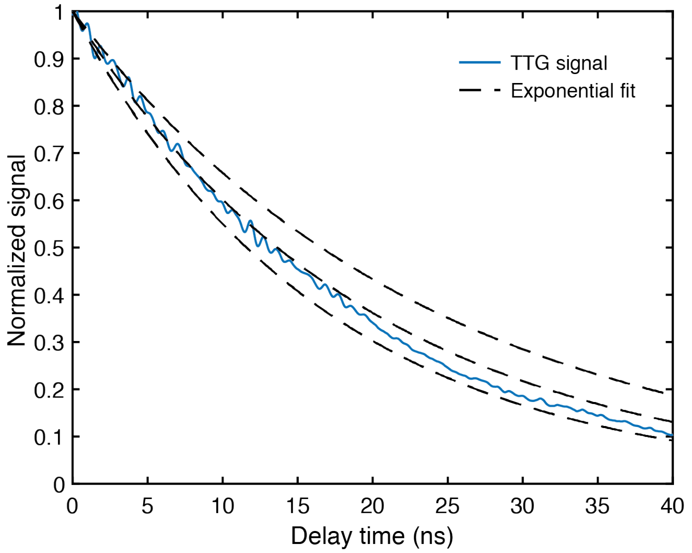

Supplementary Figure 3. Illustration of fitting the TTG signal with photoexcitation using single exponential curves (e^{-bt}). Curves with different values of b are shown as dashed lines ($b = 0.060\ \mathrm{ns}^{-1}, 0.051\ \mathrm{ns}^{-1}, 0.042\ \mathrm{ns}^{-1}$ for curves from the lowest to the highest, respectively, corresponding to thermal conductivity values of $k = 100, 85, 70\ \mathrm{W\ m^{-1}\ K^{-1}}$ at a grating period of 9 μm) and cannot fit the entire TTG signal. It can be seen that the initial TTG signal decay is closer to an exponential decay with a smaller decay time while later the signal approaches one with a slower decay, indicating the varying thermal conductivity with time.

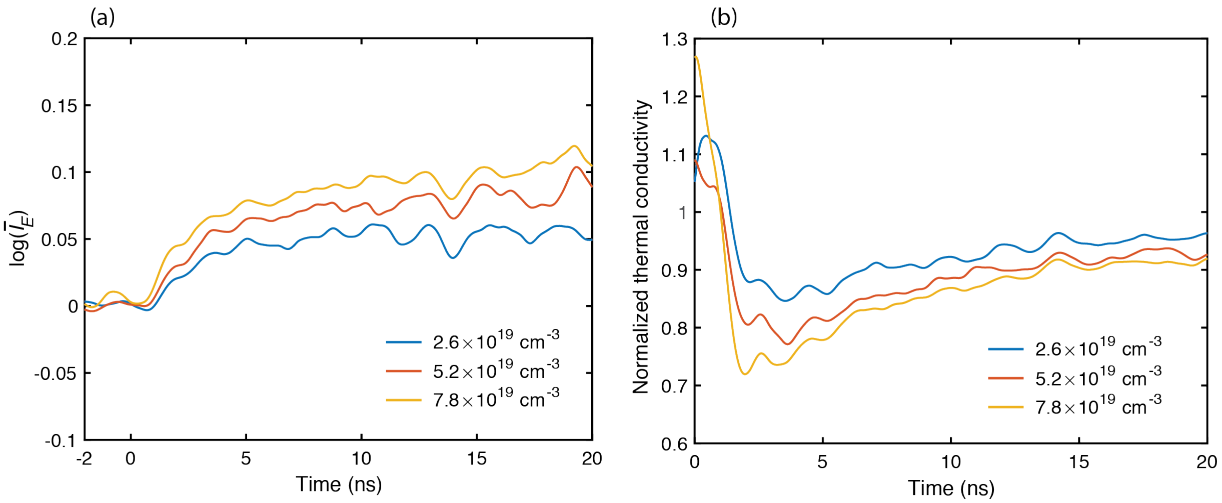

Supplementary Figure 4. Extraction of effective thermal conductivity from measurements. (a) Logarithmic ratio between the normalized TTG signals with and without photoexcitation, defined as $\log(\bar{I}_E) = \log\left(\frac{I_E(t)/I_E(0)}{I_0(t)/I_0(0)}\right)$, where the subscript E indicates the excitation pulse energy used. (b) Extracted effective thermal conductivity $k_{\mathrm{eff},E}(t)$ based on $k_{\mathrm{eff},E}(t) = k_{\mathrm{eff},0} - \frac{\rho c_\mathrm{p}}{2q^2 t}\log(\bar{I}_E(t))$. The quantity $\log(\bar{I}_E)$ represents the cumulative effect of electron-phonon interaction on the thermal decay from the time of excitation. With the definition of the effective thermal conductivity given in Methods, we can show that $\log(\bar{I}_E) = \frac{2q^2}{\rho c_\mathrm{p}}\int_0^t [k_{\mathrm{eff},0} - k_\mathrm{x}(\tau)]\mathrm{d}\tau$, where $k_\mathrm{x}(t)$ is the instantaneous thermal conductivity at time *t*. At later times when the effect of electron-phonon interaction diminishes, the quantity $\log(\bar{I}_E)$ approaches a constant, as shown in (a).

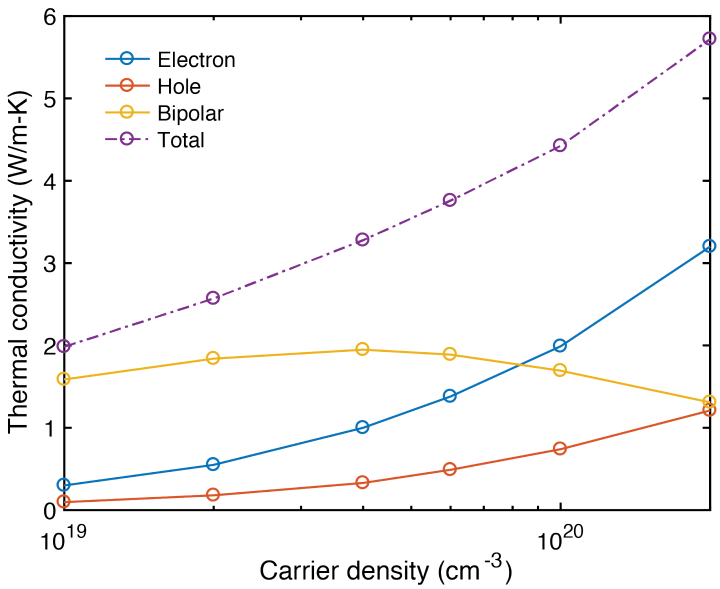

Supplementary Figure 5. Simulated electronic and bipolar contributions to the thermal conductivity from first principles calculations. At a carrier density of 1×10^{20} cm^{-3}, the total contributions from electrons and holes sum up to ~4.5 W m^{-1} K^{-1}. Details of the calculation are given in Methods.

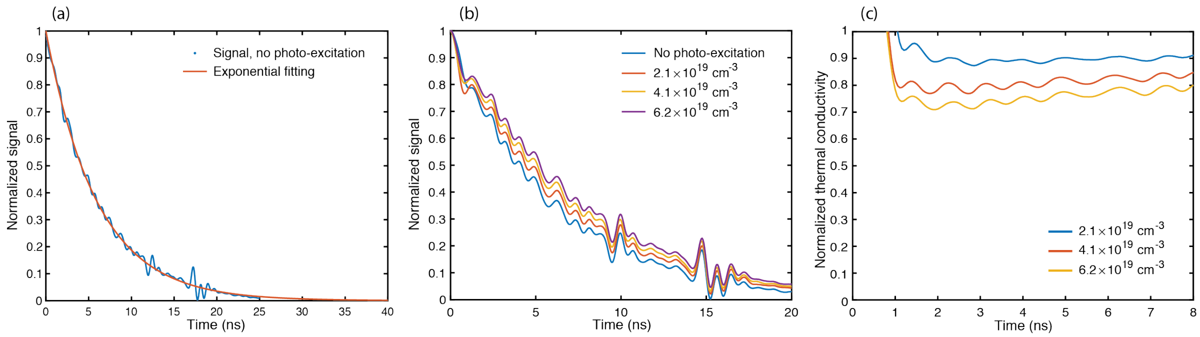

Supplementary Figure 6. Transient thermal grating measurements with photo-excitation at a smaller grating period ($L = 5\mu\mathrm{m}$). (a) Exponential fitting of the signal without photo-excitation gives a thermal conductivity of $\sim 85\ \mathrm{W\ m^{-1}\ K^{-1}}$. This value is lower than that obtained with longer grating period as some phonons have mean free paths comparable or longer than the grating period. (b) Transient thermal decay under different levels of photo-excitations, clearly showing that the photo-excited carriers lead to a slowdown of the thermal decay. (c) Effective thermal conductivities normalized to that of the silicon membrane at this grating period. A reduction of thermal conductivity over 25% is found at a carrier concentration of $6.2 \times 10^{19}\ \mathrm{cm^{-3}}$.

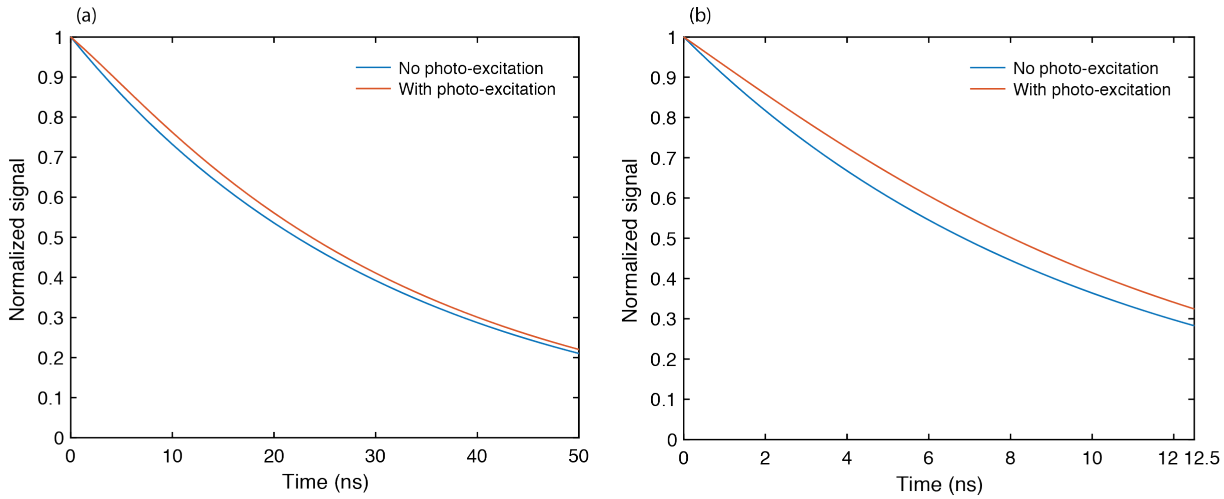

Supplementary Figure 7. Illustration of the impact of photo-excited carriers on the thermal decay at different grating periods: (a) $L = 9\mu\text{m}$, and (b) $L = 5\mu\text{m}$, using a simplified one-dimensional heat diffusion model. In this model, the TTG signal is assumed to be proportional to $|A|^2$, where A represents the magnitude of the temperature variation as described in Methods (the average temperature along membrane thickness follows $\bar{T} = A(t)\sin(qx)$). In solving the heat diffusion equation $\frac{dA}{dt} = -\frac{q^2 k_\text{x}(t)}{\rho c_\text{p}} A$, the time-varying thermal conductivity is assumed to have a reduced value (k_1) at time zero, and gradually returns to its original one without excitation (k_0), with a characteristic time τ_0 given by the carrier recombination: $k_\text{x}(t) = k_0 + (k_1 - k_0)e^{-\frac{t}{\tau_0}}$. Here we take $k_0 = 105\ \text{W m}^{-1}\ \text{K}^{-1}$, $k_1 = 75\ \text{W m}^{-1}\ \text{K}^{-1}$, and $\tau_0 = 5$ ns corresponding to the experimental condition. This model serves only to qualitatively illustrate the expected changes on the thermal decay due to photo-excited carriers. The simulated TTG signal agrees generally well with the experimental data (comparing (a) to Fig. 2a, and (b) to Supplementary Fig. 2b). The larger deviation observed at a smaller grating period is because the characteristic decay time of the heat diffusion is less than or comparable with the carrier recombination time. Therefore, the photo-excited carriers have sufficient time to impact during the major course of the thermal decay, thereby leading to more pronounced changes.

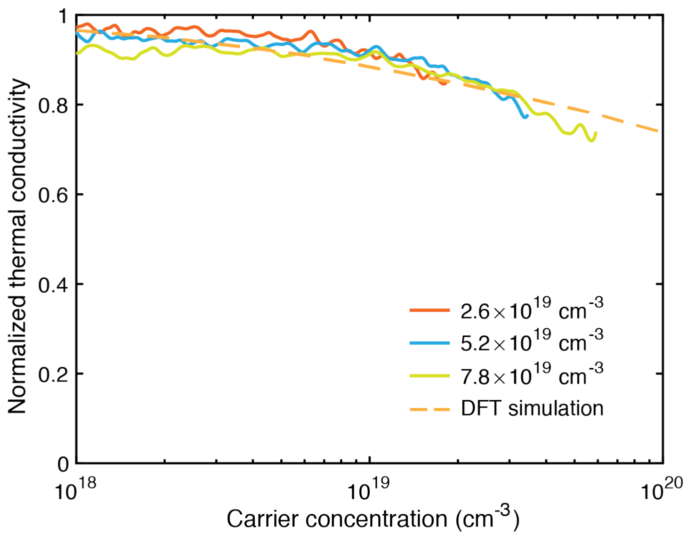

Supplementary Figure 8. Normalized thermal conductivity with carrier concentration, obtained by replotting the effective thermal conductivities in Fig. 3a (normalized to the thermal conductivity of the silicon membrane) in terms of the instantaneous carrier density during the course of the transient thermal decay. The carrier density at any given time $n(t)$ is assumed to follow an exponential decay: $n(t) = n_0 e^{-t/\tau}$, where n_0 is the initially generated carrier density (as given in the figure legend), and τ is the characteristic recombination lifetime, obtained by fitting the free carrier absorption data in Fig. S2a and interpolating the resulting decay time constants. The fitting range has been chosen to be from 0 ns to 50 ns for all excitation powers to represent the average decay rate. The characteristic recombination lifetimes thus obtained, for the three carrier densities shown here from the lowest to the highest, are 10.2 ns, 9.1 ns, and 7.2 ns, respectively.

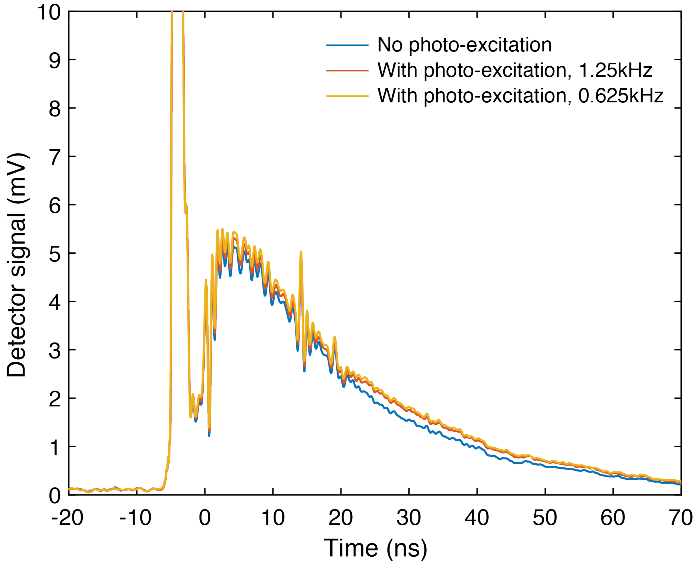

Supplementary Figure 9. Test of the effect of steady state heating on the transient thermal decay. The difference between the signals at different repetition rates is mostly due to the laser power drift. The small variation in the laser power creates variation in the TTG signal, and can be seen before the excitation pulse arrives. Transient thermal decay under photo-excitation is monitored with two different repetition rates (1.25 kHz and 0.625 kHz) that differ by a factor of two. The magnitude of steady state heating therefore should also differ by approximately a factor of two. The overlap between these two signals indicate that the steady state heating has minimum impact on the observed changes in the transient thermal decay.

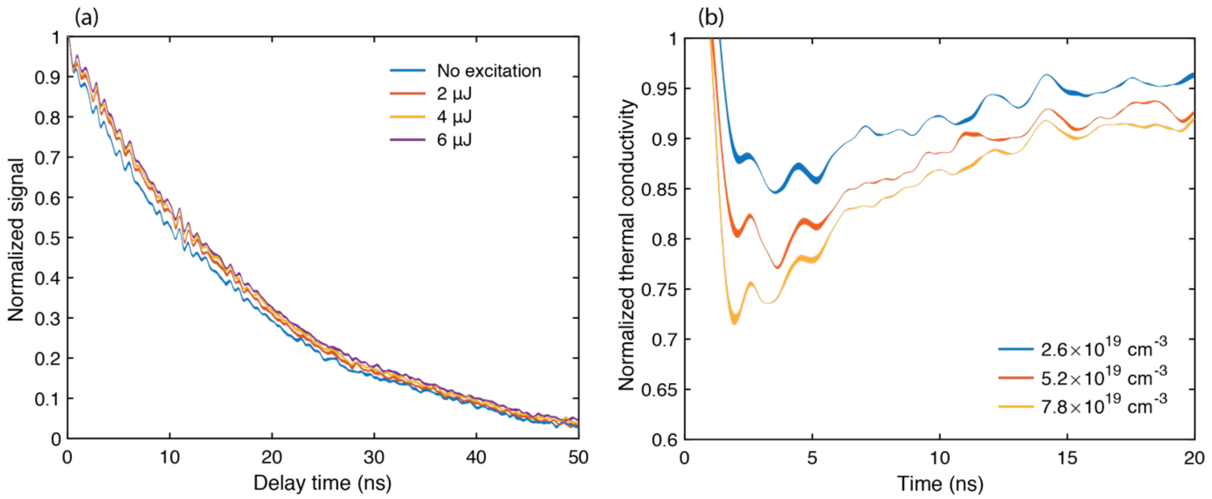

Supplementary Figure 10. 95% Confidence intervals of (a) TTG signals and (b) extracted effective thermal conductivities at different excitation fluences, corresponding to data in Fig. 2(a-b) from the main text. The 95% confidence intervals are obtained by calculating the standard error of the sample mean at each time point, and drawn as shaded regions. Owing to the large number of data traces used for the average and the resulting small standard error in the TTG signals, the uncertainty in the extracted effective thermal conductivity for a single measurement is generally less than 2%.

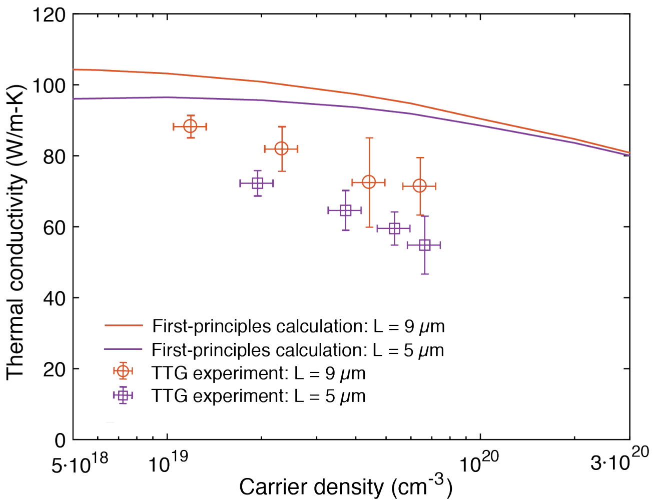

Supplementary Figure 11. Direct comparison between experimentally extracted effective thermal conductivities at different grating periods and first-principles-computed thermal conductivity with respect to the carrier density. The calculation of the thermal conductivity in this figure also considers the size effect due to the finite grating period, using an approximate solution based on the variational approach (see Supplementary Note 2). The experimental thermal conductivities lie below the theoretical curve possibly because the sample contains defects that have further reduced the thermal conductivity. The thermal conductivities obtained with a grating period of 5 μm are smaller than those at 9 μm because the smaller grating period imposes a stronger size effect on the heat transport. Nonetheless, the decreasing trend in the thermal conductivity as the carrier density increases agree between experiment and simulation.

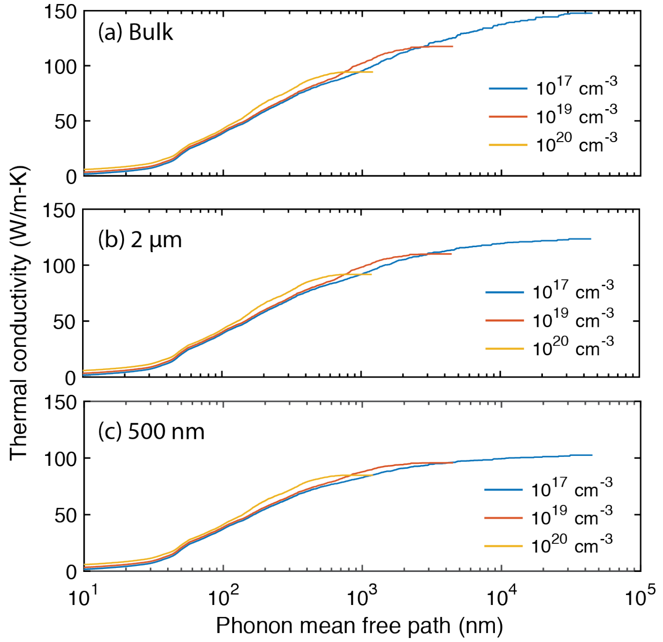

Supplementary Figure 12. Computed accumulated thermal conductivity with respect to the phonon mean free path at different carrier densities, for (a) bulk silicon, (b) silicon membrane with 2 μm thickness, and (c) silicon membrane with 500 nm thickness. The thermal conductivity includes both lattice and electronic contributions as in Fig. 3b. Carrier density of $10^{17}\ \mathrm{cm}^{-3}$ is close to the intrinsic case with phonon mean free paths spanning from nanometer to micrometer. For a bulk silicon sample at the carrier density of $10^{19}\ \mathrm{cm}^{-3}$, carriers dominantly scatter phonons with mean free paths longer than around $2\ \mu\mathrm{m}$, leading to a thermal conductivity reduction of about 20% (a). At $10^{20}\ \mathrm{cm}^{-3}$, carriers dominantly affect phonons with mean free paths longer than around $1\ \mu\mathrm{m}$, with nearly 40% reduction in thermal conductivity (a). For a silicon membrane with 2 μm thickness, phonons with mean free paths longer than 1 μm still contribute substantially (about 25%) to the thermal conductivity. As a result, the thermal conductivity reduction at carrier densities of $10^{19}\ \mathrm{cm}^{-3}$ and $10^{20}\ \mathrm{cm}^{-3}$ is about 10% and 30% respectively. If the silicon film thickness is further reduced to 500 nm, phonons are now strongly scattered by boundaries. In such case, the effect of increasing the carrier density is smaller. The thermal conductivity reduction at the carrier density of $10^{19}\ \mathrm{cm}^{-3}$ and $10^{20}\ \mathrm{cm}^{-3}$ is only about 7% and 20% respectively.

Supplementary Note 1. Derivation of phonon-boundary scattering

Here we provide a derivation of the phonon-boundary scattering term used in Methods to include the boundary scattering effect in the silicon membrane. Typically, the thermal conductivity in bulk materials can be derived from Boltzmann transport equation, with the phonon distribution function being a homogeneous function. For thin films, the distribution functions vary spatially and have to be solved from the Boltzmann transport equation combined with proper boundary conditions. Under the relaxation time approximation at steady state, the phonon Boltzmann transport equation reads:

$$\mathbf{v} \cdot \nabla_r f_{q\lambda}(\boldsymbol{r}) = -\frac{f_{q\lambda} - f_{q\lambda,0}}{\tau_q} \tag{1}$$

We consider a small deviation of the distribution function from the equilibrium: $f_{q\lambda}(\boldsymbol{r}) = f_{q\lambda,0}(T(\boldsymbol{r})) + f_{q\lambda,1}(\boldsymbol{r})$. For a thin film with a temperature gradient in the *x*-direction, the spatial derivative in the *y* direction is zero. Furthermore, the gradient of the equilibrium distribution in the *z* direction is also zero. Lastly, for the gradient of the distribution function in the *x* direction, we ignore the deviation part as the major contribution will be from the equilibrium part due to the temperature gradient. All these lead to

$$v_x \frac{\partial f_{q\lambda,0}(T(x))}{\partial T}\frac{\partial T}{\partial x} + v_z \frac{\partial f_{q\lambda,1}(x,z)}{\partial z} = -\frac{f_{q\lambda,1}}{\tau_{q\lambda}} \tag{2}$$

This boundary condition can be obtained by considering the phonon reflection for each phonon mode at the boundary. If we denote $z = 0$ as the bottom surface of the thin film, phonons arriving at this surface for phonon mode (q, λ) (assuming this phonon is going towards the bottom surface, or $v_z < 0$) have a distribution function of $f_{q\lambda,0}(z = 0) + f_{q\lambda,1}(z = 0)$. We assume the effect of the boundary is only to re-direct the phonons and does not change their energies (elastic scattering). There is also a probability that the phonons will be specularly reflected, denoted by the specularity ratio p_s. Based on this, part of the phonons (q, λ) arriving at the boundary will directly contribute to the distribution function at $(\bar{q}, \lambda)$, where $\bar{q}$ is along the specular reflection direction from phonon wavevector q. Another part of the contribution to the distribution function at $(\bar{q}, \lambda)$, which is $f_{\bar{q}\lambda,0}(z = 0) + f_{\bar{q}\lambda,1}(z = 0)$, is the diffusively scattered portion. Because the boundary scattering is elastic and the equilibrium distribution function only depends on phonon energy and temperature, we have $f_{q\lambda,0}(z = 0) = f_{\bar{q}\lambda,0}(z = 0)$ and denote this as $f_0(\omega(q\lambda), T(z = 0))$. Because in diffusive scatterings phonons are uniformly scattered into all angles, the diffusively scattered contribution to the distribution function at $(\bar{q}, \lambda)$ is then $(1 - p_s) f_0(\omega(q\lambda), T(z = 0))$. Adding up all contributions we obtain

$$p_s\left[f_{q\lambda,0}(z = 0) + f_{q\lambda,1}(z = 0)\right] + (1 - p_s) f_0\big(\omega(q\lambda), T(z = 0)\big) = f_{\bar{q}\lambda,0}(z = 0) + f_{\bar{q}\lambda,1}(z = 0)$$

The equilibrium distribution functions cancel as they have the same value. The boundary condition is now reduced to

$$p_s f_{q\lambda,1}(z=0) = f_{\bar{q}\lambda,1}(z=0) \tag{3}$$

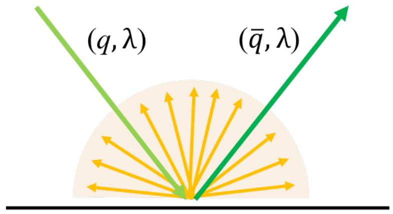

Illustration of boundary condition for phonon distribution function. The contribution to the phonon modes going outwards with wave vector $\bar{q}$ comes partly from specularly reflected phonons, and partly from diffusely scattered phonons.

We also note that the distribution functions for phonons moving upwards and downwards are related by the symmetry: $f_{q\lambda,1}(z) = f_{\bar{q}\lambda,1}(d-z)$. Combining all above, one can solve for the phonon distribution function at any wave vector and position. For phonons moving upwards ($v_z > 0$), the solution is (for phonons moving dowards the distribution function is obtained by symmetry)

$$f_{q\lambda,1} = -\tau_{q\lambda} v_x \frac{\partial f_0}{\partial T}\frac{\partial T}{\partial x}\left[1 - \frac{1-p_s}{1-p_s e^{-\frac{z}{\tau_{q\lambda} v_z}}} e^{-\frac{z}{\tau_{q\lambda} v_z}}\right] \tag{4}$$

The phonon distribution varies over the film thickness due to the boundary scatterings. The averaged phonon distribution over the film thickness is

$$\overline{f_{q\lambda,1}} = \frac{1}{d}\int_0^d dz\, f_{q\lambda,1} = -\tau_{q\lambda} v_x \frac{\partial f_0}{\partial T}\frac{\partial T}{\partial x}\left[1 - \frac{\tau_{q\lambda} v_z}{d}\frac{(1-p_s)\left(1-e^{-\frac{d}{\tau_{q\lambda} v_z}}\right)}{1-p_s e^{-\frac{d}{\tau_{q\lambda} v_z}}}\right] \tag{5}$$

With this one can then evaluate the heat flux by integrating the phonon distribution over the thickness of the thin film:

$$J_x = \frac{1}{V}\sum_{q\lambda} \hbar\omega v_x \overline{f_{q\lambda,1}} \tag{6}$$

Dividing the heat flux by the temperature gradient one obtains the thermal conductivity. For bulk samples, the phonon distribution $f_{q\lambda,1}^{\mathrm{bulk}} = -\tau_{q\lambda} v_x \frac{\partial f_0}{\partial T}\frac{\partial T}{\partial x}$ (can be obtained by taking the thickness to the infinite) does not depend on position and the resulting thermal conductivity takes the form of $k_{\mathrm{bulk}} = \frac{1}{V}\sum_{q\lambda} \hbar\omega v_x^2 \tau_{q\lambda} \frac{\partial f_0}{\partial T}$. Comparing the averaged phonon distribution in the thin film with that in the bulk geometry, we see that the thermal conductivity of a thin film can be written in a form similar to that of the bulk thermal conductivity by adding a reduction factor due to the boundary scatterings for each phonon mode

$$k_{\text{film}} = \frac{1}{V}\sum_{q\lambda} \hbar\omega v_x^2 \tau_{q\lambda} \frac{\partial f_0}{\partial T} S_{q\lambda} \tag{7}$$

where the mode-dependent reduction factor $S_{q\lambda}$ is defined by (in the above derivation we only specifically write out the formula for upwards moving phonons, here the formula is valid for all phonons by using the absolute value of the phonon group velocity)

$$S_{q\lambda} = \left[1 - \frac{\tau_{q\lambda}|v_z|}{d} \frac{(1-p_s)(1-e^{-\frac{d}{\tau_{q\lambda}|v_z|}})}{1-p_s e^{-\frac{d}{\tau_{q\lambda}|v_z|}}}\right] \tag{8}$$

For normal thin films, the relaxation times ($\tau_{q\lambda}$) are caused by phonon-phonon scatterings ($\tau_{q\lambda}^{\text{ph-ph}}$). With free carriers, the electron-phonon interaction introduces an additional scattering mechanism, as characterized by a different phonon relaxation time ($\tau_{q\lambda}^{\text{ph-e}}$). Under photo-excitation, the phonon relaxation times are calculated according to the Matthiessen's rule: $\frac{1}{\tau_{q\lambda}} = \frac{1}{\tau_{q\lambda}^{\text{ph-ph}}} + \frac{1}{\tau_{q\lambda}^{\text{ph-e}}}$, and the reduction factor further considers the phonon-boundary scatterings.

Supplementary Note 2. Transient thermal grating of thin films

Throughout the article, we have only considered phonon boundary scattering when calculating the thermal conductivity and do not consider the size effect due to the finite grating period, because the grating periods (9 μm and 5 μm) used are generally larger than the sample thickness (2 μm). Here, we discuss a method that allows consideration of the size effect due to phonon-boundary scattering and finite grating period simultaneously.

A rigorous solution of the Boltzmann transport equation for the transient thermal grating decay in a thin film would require expensive numerical simulations. Here we use an approximate solution based on variational approach, recently developed to solve spectral Boltzmann transport equation, which has shown good agreement with numerical simulations[1]. In brief, when the material has isotropic phonon properties (frequency, relaxation time, etc.) that do not depend on the velocity direction, the effective thermal conductivity extracted from the decay of a transient thermal grating experiment can be obtained as

$$k = \frac{\frac{1}{3}\int d\omega C_\omega v_\omega \Lambda_\omega \frac{3}{\eta_\omega^2}\left\{1-\frac{1}{\eta_\omega}\arctan(\eta_\omega)+\Psi(\eta_\omega,\mathrm{Kn}_\omega)\right\}}{\frac{1}{C}\int d\omega C_\omega\left\{\frac{1}{\eta_\omega}\arctan(\eta_\omega)-\Psi(\eta_\omega,\mathrm{Kn}_\omega)\right\}} \tag{9}$$

where C_ω is the heat capacity per unit frequency, v_ω the phonon velocity, and Λ_ω the phonon mean free path at given frequency ω. $\eta_\omega = q\Lambda_\omega$ characterizes the size effect of the finite grating period with q being the grating wave vector, while $\mathrm{Kn}_\omega = \Lambda_\omega/d$ is the Knudsen number with d being the sample thickness. The function Ψ appearing in Equation (9) is given by

$$\Psi(\eta_\omega, \mathrm{Kn}_\omega) = \psi_2 - \psi_1^2/\psi_0 \tag{10}$$

and

$$\psi_n(\eta_\omega, \mathrm{Kn}_\omega) = \int_0^1 d\phi \int_0^1 d\mu \frac{\mathrm{Kn}_\omega \mu}{\left(1 + i\eta_\omega\sqrt{1-\mu^2}\cos(2\pi\phi)\right)^n} \times \left[1 - \exp\left(-\frac{1 + i\eta_\omega\sqrt{1-\mu^2}\cos(2\pi\phi)}{\mathrm{Kn}_\omega \mu}\right)\right]$$

While practical materials do not have exactly isotropic phonon properties, this approximation is valid for materials with isotropic structure, such as silicon. Therefore, we assume the suppression functions which appear within the integral of the thermal conductivity expression (specifically, $\frac{3}{\eta_\omega^2}\left\{1 - \frac{1}{\eta_\omega}\arctan(\eta_\omega) + \Psi(\eta_\omega, \mathrm{Kn}_\omega)\right\}$ for the numerator and $\left\{\frac{1}{\eta_\omega}\arctan(\eta_\omega) - \Psi(\eta_\omega, \mathrm{Kn}_\omega)\right\}$ for the denominator) takes the same form using computed phonon properties on a discrete $\mathbf{q}$ mesh:

$$k = \frac{\frac{1}{3VN}\sum_{\mathbf{q}} \hbar\omega_q v_q^2 \tau_q \frac{\partial n_q}{\partial T}\frac{3}{\eta_q^2}\left\{1-\frac{1}{\eta_q}\arctan(\eta_q)+\Psi(\eta_q,\mathrm{Kn}_q)\right\}}{\frac{1}{C}\frac{1}{3VN}\sum_{\mathbf{q}} \hbar\omega_q \frac{\partial n_q}{\partial T}\left\{\frac{1}{\eta_q}\arctan(\eta_q)-\Psi(\eta_q,\mathrm{Kn}_q)\right\}} \tag{11}$$

where Λ_ω includes phonon-phonon and phonon-carrier scatterings. Equation (11) allows us to estimate the thermal conductivity of silicon thin film considering the size effect due to finite grating period.

Reference

1. Chiloyan, V. *et al.* Variational approach to solving the spectral Boltzmann transport equation in transient thermal grating for thin films. *Journal of Applied Physics* **120**, 025103 (2016).
